# Effective antitumor immunity against murine gliomas using dendritic cells transduced with hTERTC27 recombinant adenovirus

**DOI:** 10.3892/or.2011.1619

**Published:** 2011-12-30

**Authors:** HAN-XIAN GONG, LEI HE, XIANG-PEN LI, YI-DONG WANG, YI LI, JUN-JIAN HUANG, ZILING WANG, DAN XIE, HSIANG-FU KUNG, YING PENG

**Affiliations:** 1 Department of Neurology, Sun Yat-sen Memorial Hospital, Sun Yat-sen University, Guangzhou; 2 Department of Neurology, The People's Hospital of Hanhai, Foshan; 3 Laboratory of Tumor and Molecular Biology, Beijing Institute of Biotechnology, Beijing; 4 Institute of Blood Transfusion; Academy of Military Medical Sciences, Beijing; 5 State Key Laboratory of Oncology in South China, Cancer Center, Sun Yat-sen University, Guangzhou; 6 Stanley Ho Center for Emerging Infectious Diseases, The Chinese University of Hong Kong, Shatin, Hong Kong SAR, P.R. China

**Keywords:** dendritic cells, cytotoxic T lymphocytes, immunotherapy, hTERTC27

## Abstract

hTERTC27, a 27-kDa hTERT C-terminal polypeptide has been demonstrated to cause hTERT-positive HeLa cell apoptosis and inhibits the growth of mouse melanoma. hTERTC27 has been associated with telomere dysfunction, regulation of gene-regulated apoptosis, the cell cycle and activation of natural killer (NK) cells, but its mechanism of action is not fully understood. Here, we report that dendritic cells (DCs) transduced with hTERTC27 can increase T-cell proliferation, and augment the concentration of interleukin-2 (IL-2) and interferon-γ (IFN-γ) in the supernatants of T cells. It can also induce antigen-specific cytotoxic T lymphocytes (CTL) against glioma cells *in vitro*. Moreover, hTERTC27 gene-transduced DCs exhibit a very potent cytotoxicity to glioma cells *in vivo*. It could prolong the survival time and inhibit the growth of glioma-bearing mice. These data suggest that hTERTC27 gene-transduced DCs can efficiently enhance immunity against gliomas *in vitro* and *in vivo*.

## Introduction

The presentation of tumor-associated antigen (TAA) by professional antigen presenting cells (APC), especially dendritic cells (DCs) play an essential role in antitumor effects *in vitro* and *in vivo* (1). DCs are believed to be the most potent professional antigen-presenting cells (2,3). They can take up antigen efficiently, and present the antigen on their surface in association with major histocompatibility complex (MHC) molecules stimulating naive T cells to proliferate and differentiate (4–6). Therefore, the investigation of DCs-based vaccines in cancer therapy has recently received much attention. Different strategies have been developed to load DCs with TAA, including synthetic peptides derived from the known antigens (7), tumor lysates (8), tumor RNA (9) and dying tumor cells (10) to induce antigen-specific immune responses. It has been reported that the endogenous processing and presentation of TAA peptides may be more efficient for cell surface presentation than the exogenous loading of synthetic TAA peptides (11).

Telomerase is a unique ribonucleoprotein that mediates RNA-dependent synthesis of telomeric DNA, the distal ends of eukaryotic chromosomes that stabilize the chromosomes during replication (12). Telomerase is active in more than 85% of human cancers and some stem cells but repressed in most normal human somatic tissue (13,14). Human telomerase reverse transcriptase (hTERT) is the rate-limiting component of telomerase (15). In cells where telomerase is activated, hTERT synthesizes a TTAGGG sequence from the RNA template that is then added to the end of the shortening chromosome (16), thus saving the cells from death. The above mechanism is exploited by tumour cells to maintain their immortality (14,17). The widespread expression of telomerase in cancer, coupled with the critical role of hTERT in the telomerase complex, suggests that hTERT maybe used as a universal TAA. Furthermore, there is increasing evidence that peptides derived from the protein of hTERT could been specifically recognized by CD8^+^ and CD4^+^ T lymphocytes (18).

hTERTC27 (C27) is an artificially derived 27 kDa C-terminal polypeptide fragment of human TERT. It has previously been demonstrated that overexpression of hTERTC27 in HeLa cells could reduce the tumorigenicity and suppress the growth of xenografted glioblastoma in nude mice (19). C27 can also upregulate genes that are involved in apoptosis, the cell cycle, and the immune response (20). The rAAV-/rAdv-hTERTC27 viral cocktail can also activate NK cells, but not T cells, against melanoma (21). Since hTERT was identified as a universal tumor-associated antigen, we hypothesize that hTERTC27 could suppress tumor growth through the specific CTL response. In the present study, we explored whether DCs-transfected with rAd-hTERTC27-EGFP (rAd-C27 DCs) would elicit potent adaptive immunity against gliomas. Recombinant adenoviral vectors were selected in this study since others have found the adenovirus to be a highly efficient and reproducible method of gene transfer into DCs (22). We found that DCs transduced with rAd-C27 effectively induce specific cytotoxic T lymphocytes (CTL) against gliomas cells *in vitro* and *in vivo*.

## Materials and methods

### Cell culture

The glioblastoma cell line GL261 was a gift from Professor Wang (Academy of Military Medical Sciences). Cells were cultured in DMEM (Gibco, Hangzhou, China), with 10% fetal bovine serum, 100 U/ml penicillin and 100 mg/ml streptomycin (Gibco). The cells were maintained at 37°C in 5% CO_2_ cultured and passaged at weekly intervals.

### DC generation from mouse bone marrow

DCs from mouse bone marrow were generated as previously described (23,24). In brief, bone marrow was flushed from the tibias and femurs of C57BL/6 mice (Laboratory Animal Center, Sun Yat-sen University, China) and depleted of erythrocytes with commercial lysis buffer (Sigma, St. Louis, MO, USA). The cells were washed twice in serum-free RPMI-1640 medium and cultured in a 6-well plate at 5×10^6^ cells/well with RPMI-1640 medium containing 10 ng/ml recombinant mouse GM-CSF (R&D Systems, Inc., USA) and 10 ng/ml recombinant mouse IL-4 (R&D Systems). On Days 3 and 5, half of the media were refreshed and fresh cytokine-containing mGM-CSF and mIL-4 media were added. On Day 6, 200 ng/ml LPS was added to the media. On Day 7, non-adherent cells obtained from these cultures were considered mature bone marrow-derived DCs.

### Flow cytometric analysis

DCs were collected and resuspended in PBS. Cells were immunostained with fluorescein isothiocyanate (FITC)-conjugated anti-mouse MHC-II, and phycoerythrin (PE)-conjugated anti-mouse CD80, CD86 antibodies (eBioscience, USA). Corresponding FITC immunoglobulin G (IgG) isotype control antibody (eBioscience) was used. A total of 1×10^6^ cells were incubated half an hour at 4°C with antibodies. The cells were then washed twice with PBS resuspended, and analyzed on a FACScan (Becton-Dickinson, USA).

### Recombinant adenovirus-mediated gene transfer

Transduction of mouse mature DCs with rAd vector was conducted in 6-well plates with 1×10^6^ DCs per well in a 2-ml volume of RPMI-1640 medium containing 10% FBS. Viruses were added to the wells at a multiplicity of infection (MOI) of 200 and the DCs were harvested after 24 h of incubation.

### Western blot analysis

For western blot analysis, proteins in the cell extracts were separated by 10% sodium dodecyl sulphate polyacrylamide gel electrophoresis (SDS-PAGE) and then transferred onto a nitrocellulose membrane. The membrane was incubated with 5% non-fat milk in PBS and then with anti-hTERT antibody (Abcam, Hong Kong, China) overnight at 4°C. After washing, the membranes were incubated with an alkaline phosphatase-conjugated goat anti-rabbit IgG antibody (DingGuo, Beijing, China) for 1 h at room temperature. Immunoreactive bands were detected using the ECL western blot analysis system.

### In vitro experimentation

#### Mixed lymphocyte reaction (MLR)

Briefly, for preparation of T lymphocytes, spleens of C57BL/6 mice were removed aseptically, passed over nylon wool with their purity determined by FACS and prepared for the following experiments. The allogeneic T cells were mixed with 1×10^5^ DCs transduced with rAd-C27 (rAd-C27 DCs), DCs transduced with rAd-EGFP (rAd-EGFP DCs) and normal control DCs in a well of flat-bottomed 96-well plates in 200 μl of RPMI-1640 containing 10% FCS, and cultured at 37°C for 3 days. DCs were used as the stimulator (S) and T cells were used as the responder cells (R) with the S/R ratio varying from 1:5 to 1:40. CCK8 (20 μl) (Dojindo, Japan) was added into each well at 6 h before termination of the incubation. Subsequently, the absorbance values (at 450 nm) were recorded on the culture medium of each sample using a Bio-Rad microplate reader (Bio-Rad Laboratories, Hercules, CA, USA).

#### Cytotoxicity assays

T cells were co-cultured with rAd-C27 DCs, rAd-EGFP DCs and normal control DCs in a 24-well tissue culture plate in a 1-ml complete RPMI-1640 medium at 37°C in 5% CO_2_ for 72 h for the cytotoxic T lymphocytes (CTL). Then the CTLs were collected and used as the effector cells in CTL assays against U87 cells. The U87 cells, as the target cells, were placed in 96-well tissue culture plates at 1×10^4^ cells/well and co-cultured with the effector cells (CTL) at the (effect/target) ratio of 5:1, 20:1 and 40:1, at 37°C in 5% CO_2_. The cytotoxic activities were determined by CCK8.

#### ELISA

cells were co-cultured with Ad-C27 DCs, rAd-EGFP DCs and normal control DCs (S/R ratio, 1:10) in a 24-well tissue culture plate in 1 ml complete RPMI-1640 medium at 37°C in 5% CO_2_ for 3 days. The cell culture supernatant was harvested. IL-2 and IFN-γ in the supernatant was determined with ELISA using anti-human IL-2 and IFN-γ purified monoclonal antibody and biotin-labeled anti-human IL-2 and IFN-γ monoclonal antibody.

### In vivo experimentation

#### Murine brain tumor model and intracerebral injection of DCs

Female C57BL/6 mice were anesthetized with an intraperitoneal injection of 4% chloral hydrate and fixed in a stereotactic head frame (Huaibei Zhenghua Instruments Co., Anhei, China). A midline scalp incision was made and the bregma was identified. Stereotactic coordinates were measured (2.0 mm lateral, and 1.2 mm anterior to the bregma) for implantation of cells into the deep frontal white matter. A burr hole was drilled at this point and 1×10^5^ GL261 cells suspended in 2.5 μl of PBS were injected through a Hamilton syringe (Shanghai Libao Instruments Co., Shanghai, China) with a fixed, 25-gauge needle at a depth of 3.0 mm relative to the dura mater. Injections were performed at 1 μl/min. The needle was withdrawn and the incision sutured. On Day 3 and Day 10 after the tumor implantation, the mice were injected with 1×10^5^ rAd-C27 DCs, rAd-EGFP DCs or DCs suspended in 2.5 μl of PBS in the same location. The animals were monitored daily after treatment and the survival time of each mouse was recorded.

#### CTL activity assay

Splenocytes were harvested as previously described and pooled from two mice per group on Day 7 after the second intracerebra injection of rAd-C27 DCs, rAd-EGFP DCs or DCs. These T cells (2×10^7^) were restimulated *in vitro* with 1×10^6^ GL26 cells which were treated with 150 μg/ml mitomycin C at 37°C for 1 h beforehand. Then the mixed cells were co-cultured for 5 days in the presence of 20 IU/ml recombinant human IL-2. GL261 cells (3×10^4^) as target cells were incubated in a 96-well plate at 37°C for 12 h. The above T cells used as effector cells were co-cultured with GL26 cells at the effector/target ratios of 5:1, 20:1 and 40:1, at 37°C in 5% CO_2_. The cytotoxic activities were determined by CCK8.

#### Histology

On Day 21 after tumor implantation, two mice from each group were euthanized to obtain brain tissues. These tissues were stained with hematoxylin and eosin (H&E) in order to clearly display the tumor outline. The tumor volume (mm^3^) was calculated using the formula of π/6xa2xb where a is width and b is length.

#### Statistical analysis

Data were analyzed using χ^2^ analysis. The *in vivo* anticancer effect of different treatments was assessed by plotting survival curves according to the Kaplan-Meier method, and groups were compared using the log-rank test. Differences were considered statistically significant when the P-value was <0.05. All statistical analyses were carried out with SPSS 13.0 software.

## Results

### Morphological and phenotypic characteristics of mouse bone marrow-derived DCs

**Figure 1:**
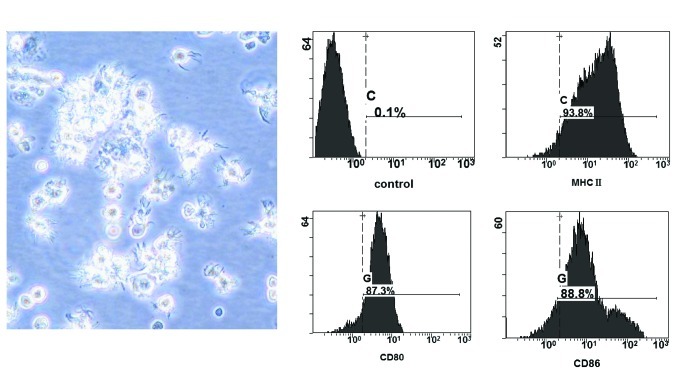
Cells derived from mouse bone marrow exhibited typical morphological and phenotypic characteristics of mature DCs. (A) Photomicrograph of DCs (original magnification, ×200). After 7 days of culture, the majority of the cells displayed the pricking and dendritic eminences on the cell surface. (B) The phenotype of the cells was analyzed by flow cytometry for the expression of the surface markers. These cells expressed high levels of CD80, CD86 and MHC-II.

On Day 7 of cell culture, mature DCs displaying typical morphological characteristics were harvested from monocytes cultured in medium containing mGM-CSF, mIL-4 and LPS. When viewed by phase contrast microscopy, these mature cells were suspended together, exhibited an irregular cell shape, and displayed the pricking and dendritic eminences on their surfaces (Fig. 1A). The phenotype of the mature DCs was analyzed using FACS. The results showed that these mature DCs expressed high levels of CD80 (87.3%), CD86 (88.8%) and MHC-II (93.8%) (Fig. 1B). The results demonstrated the successful preparation of DCs from the bone marrow of mice to be used for subsequent experiments.

### Infection of DCs with adenovirus and the identification of hTERTC27 protein expression

**Figure 2:**
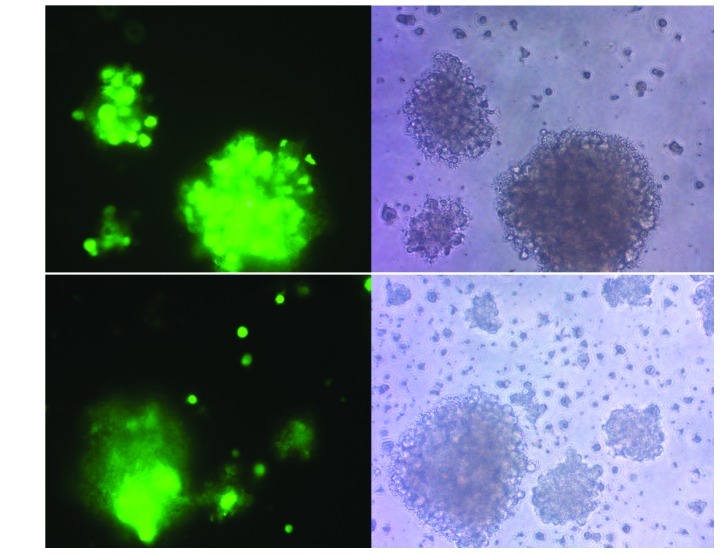
Adenovirus-mediated gene transfer was highly efficient. Strong EGFP expression in DCs transfected with the adenovirus vector were observed under phase contrast microscope and fluorescence microscope (×100). (A) DCs transfected with rAd-C27. (B) DCs transfected with rAd-EGFP.

**Figure 3:**
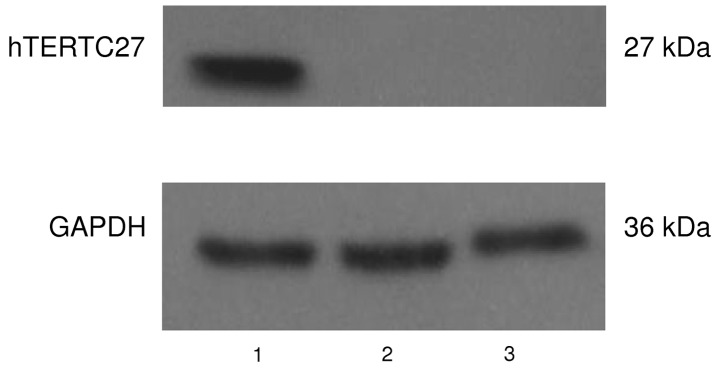
rAd-C27 DCs expressed hTERTC27 protein. The results show that only the DCs infected with rAd-C27 could express hTERTC27 protein. Lane 1, DCs transduced with rAd-C27; lane 2, DCs transduced with rAd-EGFP; lane 3, DCs.

DCs derived from mouse bone marrow were cultured in the previously-defined medium containing mGM-CSF and mIL-4. rAd-C27 was constructed and used to infect the DCs at an MOI of 200 by the centrifugal method, which was optimized for suitable transfection efficiency and toxicity. A control vector rAd-EGFP was used to infect the DCs in parallel, which resulted in EGFP expression in about 80% of the DCs (Fig. 2A), indicating a high transduction efficiency of the adenovirus vector (Fig. 2B). The expression of the hTERTC27 protein was readily detectable in the DCs 48 h after the adenoviral infection by western blot analysis using an anti-hTERT antibody. As shown in Fig. 3, only the DCs transduced with rAd-C27 expressed hTERTC27 protein. These results suggest that hTERTC27 gene was successfully transfected into DCs and the adenovirus-mediated gene transfer was highly efficient.

### Improved proliferation of T cells

**Figure 4:**
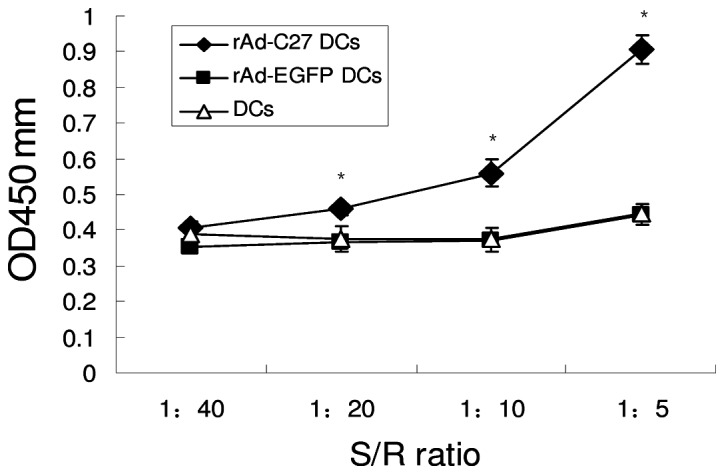
DC stimulatory ability was detected in allogeneic mixed lymphocyte reactions (MLR) at varied ratios of DCs to T cells. Allogeneic T cells were mixed with 1×10^5^ rAd-C27 DCs, rAd-EGFP DCs and normal control DCs, stimulator/responder (S/R) ratios varying from 1:5 to 1:40, and incubated for 3 days. ^*^P<0.05.

rAd-C27 and rAd-EGFP transduced DCs respectively on Day 3 after transduction were used to induce T cell proliferation. Allogeneic T cells were mixed with 1×10^5^ rAd-27 DCs, rAd-EGFP DCs and normal control DCs, at stimulator/responder ratios of 1:40, 1:20, 1:10, 1:5, and incubated for 3 days (Fig. 4). rAd-27 DCs demonstrated identical levels of stimulation which was much higher than the other two groups at S/R ratios from 1:20 to 1:5 (P<0.05), while the levels of rAd-EGFP DCs and DCs were nearly identical. These results indicate that the enhancement was related to the transgene expression and not due to adenoviral infection. Therefore, rAd-27 DCs could induce stronger proliferation of T cells.

### Induced CTL responses in vitro

**Figure 5:**
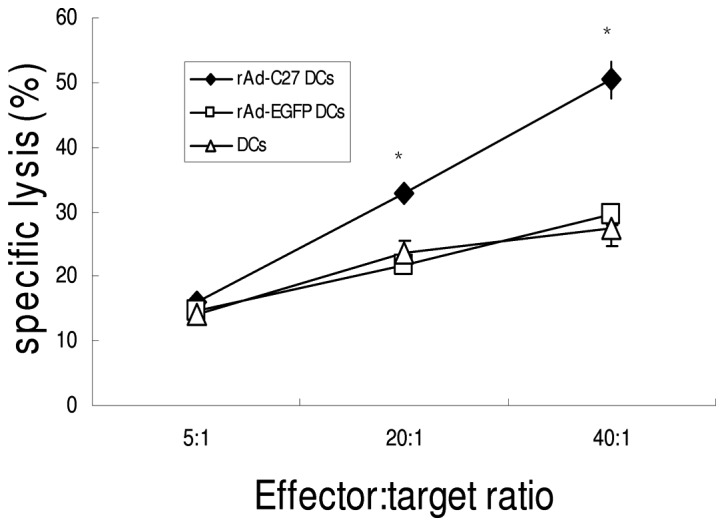
rAd-C27-DCs promote CTL generation *in vitro*. The DCs were infected with rAd-C27 at a multiplicity of infection (MOI) of 200. The percentage lysis of target cells was determined by using the CCK8 assay. The cytotoxic activity of rAd-C27 DCs was greater than that of rAd-EGFP DCs and DCs at the effector/target ratios of 20:1 and 40:1 (^*^P<0.01).

The functional capability of the CTL responding to adenovirus-infected DCs was examined by determining whether they could specifically lyse tumor cells. The T cell lines were generated from autologous mononuclear cells and were plated in 96-well plates in medium containing IL-2 (2 ng/ml). The DCs were added at varying T cell-to-DC ratios and co-cultured at 37°C in 5% CO_2_. As a result, rAd-C27 DCs could induce specific CTL activity against hTERT positive GL261 cells (Fig. 5). The cytotoxic activity of rAd-C27 DCs was 50.38±2.95% at a 40:1 effector/target ratio (E/T), while no obvious lysis by rAd-EGFP DCs or DCs was detected, even at the highest E/T ratio (29.53±1.49%, 27.53±2.71%). These results clearly demonstrate that the CTL were mainly induced by hTERTC27 peptides.

### Augmentation of the concentration of IL-2 and IFN-γ in the supernatants of T cells

**Figure 6:**
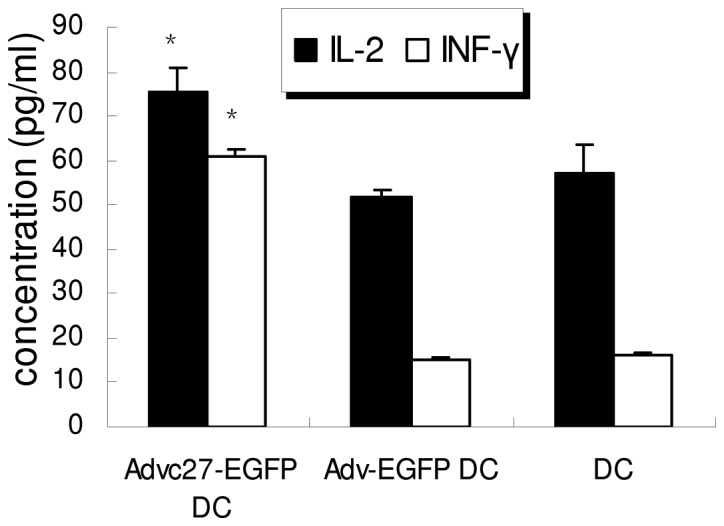
The concentration of IL-2 and IFN-γ in the supernatants of T cells were assayed with ELISA. The T cells were co-cultured with the rAd-C27 DCs, rAd-EGFP DCs and normal control DCs at a stimulator/responder ratio of 1:10. The concentration of both IL-2 and IFN-γ in T cells co-cultured with Advc27-EGFP DCs group were higher than in the other two groups. ^*^P=0.001.

The T cells were co-cultured with the rAd-C27 DCs, rAd-EGFP DCs and normal control DCs for 3 days. IL-2 and IFN-γ in the supernatant was determined with ELISA (Fig. 6). The T cells co-cultured with rAd-C27 DCs produced 75.54±5.32 pg/ml of IL-2, while IL-2 produced by rAd-EGFP DCs group and DCs group was 51.83±1.39 and 57.23±6.30 pg/ml, which both were lower than rAd-C27 DCs group (P=0.001). Also, the concentration of IFN-γ in rAd-C27 DCs group was higher than other groups. These results suggest that an hTERTC27 vaccine may increase IL-2 and IFN-γ secretion to enhance the immune response.

### Prolongation of the survival time of tumor bearing mice

**Figure 7:**
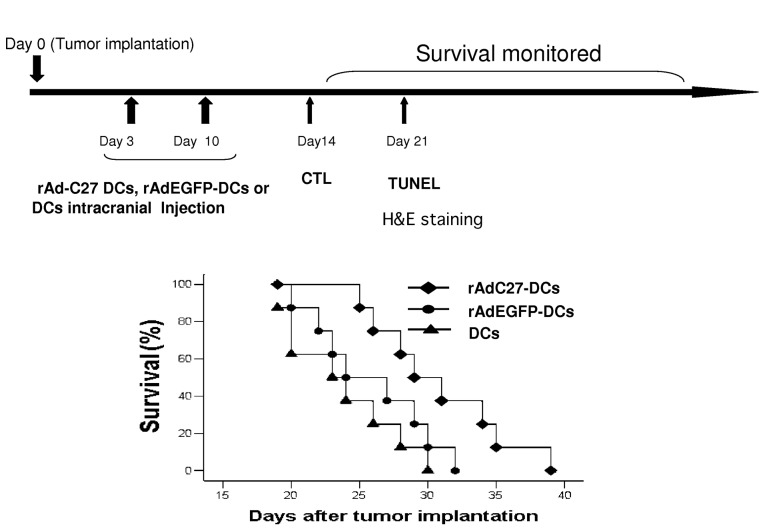
*In vivo* experiments using the intracranial tumor model. (A) The experimental schema shows tumor implantation, DCs injection, CTL, TUNEL, and survival observation time. (B) Effect of DCs on the experimental glioma in C57BL/6 mice. Kaplan-Meier survival curve of intracranial glioma-bearing C57BL/6 mice that were injected twice with rAd-C27 DCs, rAd-EGFP DCs or DCs.

To examine whether the rAd-C27 DCs provided a therapeutic benefit for brain tumor, we implemented DCs immunotherapy in an established mouse glioma model. rAd-C27 DCs, rAd-EGFP DCs or DCs (1×10^5^ each) were intracerebrally injected on Day 3 and Day 10 after the tumor implantation (Fig. 7A). The administration of Ad-C27 DCs resulted in a significantly prolonged survival compared with rAd-EGFP DCs or DCs (Fig. 7B). Approximately 50% of the rAd-C27 DCs-treated mice survived beyond an observation period of 30 days, but almost all mice treated with rAd-EGFP-DCs and DCs survived for 30 days. Statistical analysis revealed that the effect of rAd-C27 DCs was significantly different from that of rAd-EGFP DCs or DCs (P=0.005). In addition, there was no significant difference between the DC-empty and non-transduced DC treatments (P=0.307).

### Induced cytotoxicity against murine gliomas in vivo

**Figure 8:**
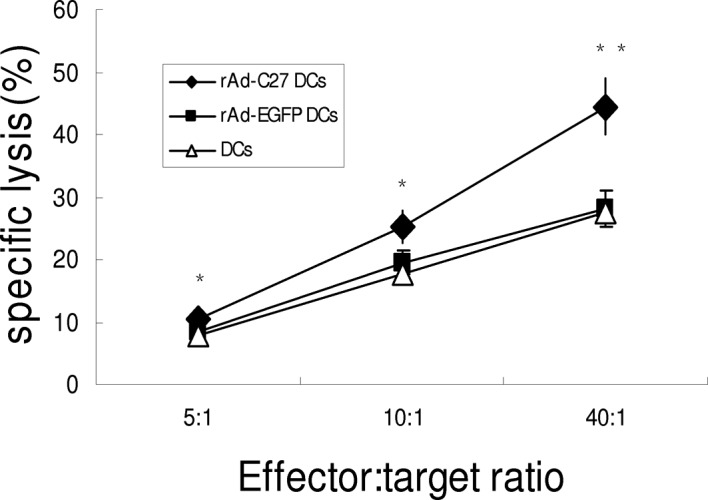
rAd-C27 DCs induced cytotoxicity against murine glioma *in vivo.* Splenocytes harvested on Day 7 after the second intracerebra injection were restimulated by GL261 and their cytotoxicity was detected by the CCK8 assay. The cytotoxicity elicited by rAd-C27 DCs was higher than the other groups at effector/target (E/T) ratios of 5:1, 10:1 and 40:1.

To further evaluate whether intratumoral injections with rAd-C27 DCs influence the induction of tumor-specific T cell responses in tumor-bearing mice, C57BL/6 mice were immunized twice, with a 7-day interval, with rAd-C27 DCs, rAd-EGFP-DCs or DCs. Mice that had received rAd-C27 DCs therapy exhibited remarkable GL261-specific CTL responses (Fig. 8). The cytotoxicity elicited by rAd-C27 DCs was much higher than the other groups at the E/T ratios of 5:1, 10:1 and to 40:1 (P<0.05). Therefore, rAd-C27 DCs could induce antigen-specific cytotoxic T lymphocytes against gliomas *in vitro* and *in vivo*.

### Inhibition of the tumor growth in C57BL mice

**Figure 9:**
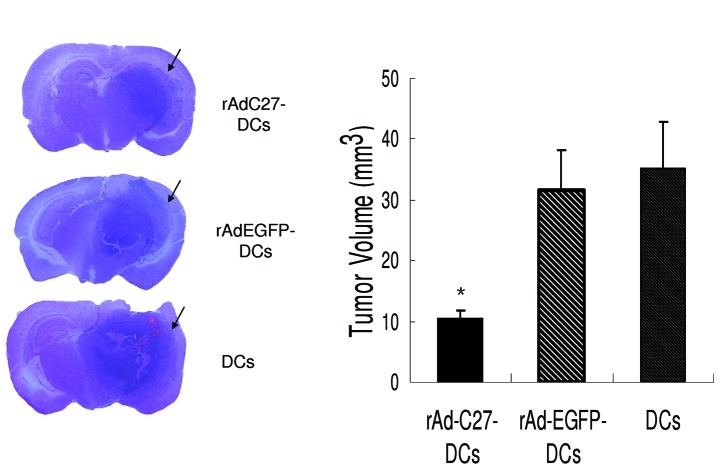
Inhibition of tumor growth in GL261 mice. On Day 21 after tumor implantation, four mice from each group were euthanized to obtain brain tissues and compare the tumor volume. (A) The brain tissues stained with hematoxylin and eosin (H&E) demarcated the tumor outline. (B) The average tumor size in different groups after GL261 glioma cells implantation.

On Day 21 after tumor implantation, four mice from each group were euthanized to obtain brain tissues and compare the tumor volume. These tissues stained with H&E clearly demarcated the tumor outline. Thus, it was verified that the tumor transplantation had been successfully performed in all tissues (Fig. 9). The average tumor size for the DC groups was 35.21±7.70 mm^3^, while it was 31.50±6.79 mm^3^ for the rAd-EGFP-DCs group. For the groups treated with rAd-C27 DCs, the average tumor sizes were 10.53±1.24 mm^3^. According to our results, the rAd-C27 DCs treatment could significantly diminish the glioma tumor size when compared with the other groups (P<0.001).

## Discussion

In this study, we have demonstrated that hTERTC27 could increase T cell proliferation, augment the concentration of IL-2 and IFN-γ in the supernatants of T cells. Furthermore, it demonstrated the ability to mount a strong and specific CTL response against the glioblastoma cells *in vitro* and *in vivo*. It could prolong the survival time and inhibit the growth of gliomas-bearing mice.

Cancer vaccine targeting tumor antigens has attracted much attention in recent years because of its higher specificity and lower toxicity than non-surgical approaches (25,26). Cancer vaccines can be categorized into peptide/protein vaccines, tumor cell vaccine, DNA vaccines, recombinant virus vaccine as well as DC vaccines (25). The ascendancy of using DC vaccines in cancer treatment is that tumor antigen(s) or DNA encoding antigen(s) will be taken up and processed easily. Here, we found that rAd-C27 DCs not only promoted T-cell proliferation, but also triggered a specific CTL response to lyse cancer cells. However, Huo *et al* (21) reported that the rAAV-/rAdv-hTERTC27 viral cocktail only revoked the NK cell response and could not induce a T lymphocyte response in the mice, which was not in accordance with our study. We presumed that the antigens contain in the rAAV-/rAdv-hTERTC27 were not sufficient to stimulate T lymphocytes, and DCs could present the antigens and augment the antigens' influence. Our presumption is supported by the following evidence. Nestle *et al* reported that peptide- or tumor lysate-loaded DCs can lead to a systemic response effective in inducing tumor regressions in the clinical study. By contrast, administration of melanoma peptides, without DCs, failed to elicit clinically significant responses (27). Therefore, DC vaccines may minimize the immune escape because of loss of antigen expression and were considered as a promising approach to tumour immunotherapy.

As a universal tumor-associated antigen, TERT is an ideal target for cancer therapy. Several immunogenic hTERT peptides have been discovered, (28) p973 and p988 were definitively contained in hTERTC27 (29,30). p988 was restricted to the MHC class I allele HLA-A^*^0201 (HLA-A2), the most frequently expressed HLA allele found among nearly 50% of Caucasians, Asians, and Hispanics and 33% of African-Americans. This evidence suggests that hTERTC27 may be utilized in tumor patients from different areas. Both p973 and p988 were demonstrated to induce specific CD8^+^ T cell responses *in vitro* or *in vivo* to lyse hTERT^+^ tumor cells. More importantly, hTERT-specific CTL lysed neither telomerase-negative normal cell nor telomerase-positive CD34^+^ hematopoietic progenitor cells (31). Activated B-cells are susceptible to hTERT-specific lysis and notably represent the only cells other than tumor cells that to date have been demonstrated to undergo hTERT-specific lysis *in vitro*. In fact, we did not find the growth difference between DCs transfected by AdvC27-EGFP and that transfected by Adv-EGFP in our study (data not shown). Therefore, the immunotherapy targeting hTERTC27 was possibly a safe and effective strategy against carcinoma.

IL-2 acts as an antitumor agent by increasing the cytolytic activity of antigen-specific cytotoxic T lymphocytes and NK cells and by increasing the gene expression responsible for encoding the lytic component of cytotoxic granules, ie, perforin and granzymes (32,33). IFN-γ, the product of Th1, CTL, and NK cells, is one of the major effector molecules in cell-mediated immunity. It plays important roles in the induction of CTL and differentiation of Th1 cells. It also leads to an increase in MHC class I and II expression and contributes to efficient antigen presentation to lymphocytes (34,35). In the present study, T cell lines co-cultured with AdvC27-EGFP-transduced DCs produced a high level of IL-2 and IFN-γ. It was possible that the high level of cytokine was one of the main reasons why AdvC27-EGFP-transduced DCs could induce a strong CTL response against the cancer cells. All in all, in this study, we have demonstrated that DCs transduced with AdvC27-EGFP are able to induce a potent antitumor immune response against hTERT-positive tumor cells, which will be instrumental for the clinical application of hTERTC27 in cancer therapy.

## Abbreviations

DCs: dendritic cells
IL-2: interleukin-2
IFN-γ: interferon-γ
CTL: cytotoxic T lymphocytes
TAA: tumor-associated antigen
APC: antigen presenting cells
hTERT: human telomerase reverse transcriptase
C27: hTERTC27
rAd: recombinant adenoviral
MLR: mixed lymphocyte reaction
NK: natural killer
